# Climate change, food, water and population health in China

**DOI:** 10.2471/BLT.15.167031

**Published:** 2016-07-13

**Authors:** Shilu Tong, Helen L Berry, Kristie Ebi, Hilary Bambrick, Wenbiao Hu, Donna Green, Elizabeth Hanna, Zhiqiang Wang, Colin D Butler

**Affiliations:** a School of Public Health and Social Work, Institute of Health and Biomedical Innovation, Queensland University of Technology, Kelvin Grove, Queensland 4159, Australia.; b Faculty of Health, University of Canberra, Canberra, Australia.; c School of Public Health, University of Washington, Seattle, United States of America.; d School of Medicine, Western Sydney University, Sydney, Australia.; e Climate Change Research Centre, University of New South Wales, Sydney, Australia.; f National Centre for Epidemiology and Population Health, Australian National University, Canberra, Australia.; g School of Medicine, University of Queensland, Brisbane, Australia.

## Abstract

Anthropogenic climate change appears to be increasing the frequency, duration and intensity of extreme weather events. Such events have already had substantial impacts on socioeconomic development and population health. Climate change’s most profound impacts are likely to be on food, health systems and water. This paper explores how climate change will affect food, human health and water in China. Projections indicate that the overall effects of climate change, land conversion and reduced water availability could reduce Chinese food production substantially – although uncertainty is inevitable in such projections. Climate change will probably have substantial impacts on water resources – e.g. changes in rainfall patterns and increases in the frequencies of droughts and floods in some areas of China. Such impacts would undoubtedly threaten population health and well-being in many communities. In the short-term, population health in China is likely to be adversely affected by increases in air temperatures and pollution. In the medium to long term, however, the indirect impacts of climate change – e.g. changes in the availability of food, shelter and water, decreased mental health and well-being and changes in the distribution and seasonality of infectious diseases – are likely to grow in importance. The potentially catastrophic consequences of climate change can only be avoided if all countries work together towards a substantial reduction in the emission of so-called greenhouse gases and a substantial increase in the global population’s resilience to the risks of climate variability and change.

## Résumé

Le changement climatique anthropique est à l'origine d'une augmentation de la fréquence, de la durée et de l'intensité de phénomènes météorologiques extrêmes. Ces phénomènes ont déjà eu des conséquences notables sur le développement socioéconomique et la santé des populations. Les effets les plus marqués du changement climatique se manifesteront certainement au niveau de l'alimentation, des systèmes sanitaires et de l'eau. Cet article examine la manière dont le changement climatique affectera l'alimentation, la santé humaine et l'eau en Chine. Selon les prévisions, les effets globaux du changement climatique, la conversion des terres et la diminution de l'eau disponible pourraient considérablement réduire la production alimentaire chinoise, bien que de telles prévisions comportent inévitablement un certain degré d'incertitude. Le changement climatique aura probablement des conséquences importantes sur les ressources en eau, avec par exemple des modifications des régimes de précipitations ou encore des sécheresses et des inondations plus fréquentes dans certaines régions de Chine. Ces effets mettront indubitablement en péril la santé et le bien-être de nombreuses communautés. À court terme, la santé des populations en Chine devrait se trouver affectée par l'augmentation des températures de l'air et de la pollution. Sur le moyen et le long terme, cependant, ce sont les effets indirects du changement climatique (par ex., changements au niveau de la disponibilité des denrées alimentaires, de l'eau et des lieux de résidence, dégradation de la santé mentale et du bien-être des personnes, changements dans la répartition et la saisonnalité des maladies infectieuses) qui devraient prendre de l'importance. Les conséquences potentiellement catastrophiques du changement climatique ne peuvent être évitées que si tous les pays œuvrent ensemble en vue de réduire sensiblement les émissions de gaz à effet de serre et d'augmenter la résilience de la population mondiale face aux risques de variabilité et de changement climatiques.

## Resumen

Parece que el cambio climático antropogénico está aumentando la frecuencia, duración e intensidad de fenómenos meteorológicos extremos. Tales fenómenos ya han afectado enormemente el desarrollo socioeconómico y la salud pública. Los efectos más profundos del cambio climático tienen posibilidades de afectar a la alimentación, los sistemas sanitarios y el agua. En este artículo se explora cómo influirá el cambio climático en la alimentación, la salud pública y el agua en China. Las previsiones indican que los efectos generales del cambio climático, la conversión del suelo y la menor disponibilidad de agua podrían reducir la producción de alimentación en China de forma sustancial, a pesar de que la incertidumbre es inevitable en dichas previsiones. Es probable que el cambio climático afecte de manera importante a los recursos de agua, por ejemplo, cambios en los patrones de las precipitaciones y aumentos de la frecuencia de las sequías y las inundaciones en algunas zonas de China. Sin duda, tales impactos amenazarán la salud pública y el bienestar de muchas comunidades. A corto plazo, la salud pública china se verá perjudicada por los aumentos de la temperatura del aire y la contaminación. Sin embargo, de medio a largo plazo, es probable que aumente la importancia de los efectos indirectos del cambio climático, como los cambios de la disponibilidad de alimentos, refugio y agua, una reducción del bienestar y la salud mental y cambios en la propagación y estacionalidad de enfermedades infecciosas. Las posibles consecuencias catastróficas del cambio climático pueden evitarse únicamente si todos los países trabajan juntos para reducir en gran medida la emisión de los conocidos gases de efecto invernadero y para aumentar la resistencia de la población mundial ante los riesgos de la variabilidad y el cambio climáticos.

## ملخص

يبدو أن التغير المناخي الناجم عن تأثيرات بشرية يؤدي إلى زيادة وتيرة حدوث الظواهر الجوية الشديدة ومدة سريانها وشدتها. وكان لمثل هذه الظواهر تأثيرات كبيرة بالفعل على التنمية الاجتماعية والاقتصادية وعلى صحة السكان. من المرجح أن تقع التأثيرات الأشد خطورة لتغير المناخ على الطعام والأنظمة الصحية والمياه. تساهم هذه المقالة البحثية في استكشاف تأثير تغير المناخ على الطعام وصحة الإنسان والمياه في الصين. تشير التوقعات إلى أن التأثيرات الإجمالية لتغير المناخ، وتبوير الأراضي الزراعية، ونقص توفير المياه يمكن أن تقلل من إنتاج الصين للغذاء بشكل كبير – بالرغم من أن مثل هذه التوقعات تحتمل قدرًا من عدم التيقن وهو أمر حتمي. من المحتمل أن يكون لتغير المناخ تأثيرات كبيرة على الموارد المائية - فعلى سبيل المثال التغيرات في أنماط هطول الأمطار وزيادة حدوث موجات الجفاف والفيضانات في بعض مناطق الصين. ومما لا شك فيه أن مثل هذه التأثيرات سوف تؤدي إلى تهديد صحة السكان وعافيتهم في العديد من المجتمعات. من المرجح أن تتأثر صحة السكان في الصين بشدة على المدى القصير بسبب الزيادة في درجات الحرارة ومعدلات التلوث. إلا أن التأثيرات غير المباشرة لتغير المناخ على المدى المتوسط إلى البعيد – على سبيل المثال التغيرات في معدل توفير الطعام والمأوى والمياه، وانخفاض مستوى الصحة العقلية والعافية، وتغير معدلات انتشار الأمراض المعدية وحدوثها بحسب الفصول – من المرجح أن تزيد أهميتها. لا يمكن تفادي العواقب الكارثية المحتملة لتغير المناخ إلا إذا تعاونت جميع البلدان وعملت معًا بهدف خفض معدلات انبعاث ما يسمى الغازات المسببة للاحتباس الحراري بنسبة كبيرة وزيادة قدرة سكان العالم على مواجهة مخاطر تقلب المناخ وتغيره.

## 摘要

由人类活动引起的气候变化导致极端天气事件的频率、持续时间和严重程度正逐渐增加。 这类事件已经对社会经济发展和人口健康产生了重大影响。 气候变化最具冲击力的影响可能是在食物、卫生系统和水源方面。 本文探究气候变化将如何影响中国的食物、人类健康和水源。 据预测，气候变化、土地流失和可用水资源减少的总体影响可能会导致中国食物产量大幅度下降——虽然此类预测中存在一定不可避免的不确定性。 气候变化可能会对水资源产生重大影响——例如，中国部分地区降水方式发生变化以及干旱与洪涝频率增加。 毫无疑问，此类影响将威胁许多社区的人口健康和幸福感。 短期来看，气温上升和污染加剧可能会对中国人口健康产生不利影响。 但从中长期来看，气候变化间接影响因素——例如可用食物、住所和水资源的变化、心理健康和幸福感的下降、传染疾病的分布和季节性的变化——可能会越来越重要。 只有各国协力合作，从本质上降低温室气体的排放，并提高全球人口对气候多样性和气候变化的适用能力，才能避免气候变化带来的潜在灾难性后果。

## Резюме

Антропогенные климатические изменения, по-видимому, способствуют увеличению частоты возникновения, продолжительности и интенсивности экстремальных погодных явлений. Такие явления уже оказали значительное влияние на социально-экономическое развитие и здоровье населения. Наиболее серьезные последствия климатических изменений, вероятно, затронут продукты питания, системы здравоохранения и воду. В данной статье изучается влияние изменения климата на продукты питания, здоровье человека и воду в Китае. Согласно результатам прогнозирования, общие последствия изменения климата, освоения земель и уменьшения доступности воды могут привести к значительному сокращению производства продуктов питания в Китае, хотя прогнозы всегда несут в себе долю неопределенности. Изменение климата, вероятно, значительно повлияет на водные ресурсы, например на режим выпадения осадков и на увеличение частоты засух и наводнений в некоторых областях Китая. Такие последствия, несомненно, поставят под угрозу здоровье и благосостояние населения во многих сообществах. В краткосрочной перспективе здоровье населения в Китае, скорее всего, подвергнется негативному воздействию повышенных температур и загрязнения воздуха. При этом в средне- и долгосрочной перспективе вероятен рост значимости косвенных последствий климатических изменений, т. е. изменения доступности продуктов питания, жилья и воды, ухудшение психического здоровья и благосостояния и изменения распределения и сезонности инфекционных заболеваний. Чтобы избежать потенциально катастрофических последствий изменения климата, все страны должны объединить свои усилия по значительному уменьшению выброса так называемых парниковых газов и значительному увеличению устойчивости мирового населения к рискам, связанным с нестабильностью и изменением климата.

## Introduction

The consequences of climate change will be felt in all of the world’s continents, countries and populations.^1^ As climate change proceeds, continuing temperature increases and changes in precipitation patterns will lead to rises in sea level and climatic zonal shifts. Climate change already appears to be increasing the frequency and intensity of droughts, floods, heatwaves, storms and other extreme weather events.^2^

At the United Nations climate summit held in Paris in late 2015, China committed to halting the growth in its emissions of so-called greenhouse gases by 2030. The peak in China’s emissions of such gases could come by 2025 – if not substantially sooner.^3^ On 16 March 2016, the Chinese Government officially approved its 13th five-year plan, the blueprint for China’s economic and social development between 2016 and 2020.^4^ The aims of this plan, which was the first to highlight environmental protection as a key priority, include 23%, 15%, and 18% reductions in water consumption, energy consumption and carbon dioxide emissions, respectively, by 2020. Another of the plan’s aims is to have good air quality – as assessed by the density of fine particle matter – on at least 80% of days per year by 2020. China’s economy needs to shift from a heavily polluting model towards a cleaner and more environmentally friendly one – and it is hoped that such a shift will lead to substantial improvements in population health and well-being.^3^^,^^4^

The most profound effects of climate change are likely to be on food, health systems and water, with cascading effects on various social systems.^5^ This paper aims to provide an overview of the potential impact of climate change on food and water in China and the consequences of that impact on the population’s health.

## Climate, food yields and nutrition

Climate change will probably affect all four key dimensions of food supply: availability, stability, access and utilization.^6^^,^^7^ It has been estimated that between 2012 and 2014, more than 2 billion people had so-called hidden hunger – that is, micronutrient insufficiency – and 795 million were undernourished.^8^^,^^9^

The world’s mean surface temperature increased by 0.8 C during the 20th century and most of that increase occurred after 1970.^1^ Changes in temperature and precipitation are already affecting regional agricultural and food production systems in many ways.^10^^,^^11^ A recent meta-analysis projected that due to climate change, mean crop yields may decline across Africa and South Asia by 8% by the 2050s.^12^ Climate change has therefore the potential to interrupt progress towards a world without hunger.^13^ China, the world’s most populous country and one of the fastest growing developing economies, is not exempt from the adverse effects of such change.^14^ China already has to import grain in an attempt to meet its food needs, and the gap between those needs and national food production is likely to increase as climate change proceeds.^15^

Mean near-surface air temperatures across China rose by 0.5–0.8 °C during the 20th century and there is evidence that this process is accelerating.^1^ Over the last 50 years, the frequency and intensity of extreme heat events in eastern China have increased and it has been suggested that anthropogenic forces have led to a 60-fold increase in the likelihood of extremely warm summers.^16^ Over the same period, droughts in northern and north-eastern China have become more severe and flooding in the middle and lower reaches of the Yangtze River and south-eastern China has intensified.^1^^,^^16^

Food security and water security in China are intertwined with a variety of anthropogenic, sociopolitical and policy factors, including air pollution, industrialization, population growth, urbanization and the increasing affluence of China’s middle class and the associated nutritional transition.^15^ Urbanization has a severe impact on agriculture and agricultural costs in China, often leading to loss of fertile land.^17^^,^^18^ Climate change, just as the current turbulence in the global energy and food markets, adds to the risks of food insecurity and is probably a factor in the keen interest shown by China and many other nations in improving agricultural fertility.^19^

Table 1 summarizes the sown areas and yields of China’s major farm crops in 2013.^20^ About half of China’s agricultural land is used to grow maize, rice or wheat.^20^^,^^21^ China holds 22% of the world’s population but only 7% of the world’s arable land.^14^ The *Fifth Assessment Report of the Intergovernmental Panel on Climate Change* indicated that climate change is already having a negative impact on agriculture and food production by adversely affecting major crops, livestock production and fisheries.^22^ It remains unclear whether China can feed its entire population adequately in the 21st century.^14^^,^^21^^,^^23^ Food insecurity remains a problem for China, especially for those living in poor and remote areas.^23^ More than 100 million farmers and their families still experience poverty and are highly vulnerable to many forms of stress. Climate change is likely to exacerbate the problems they face, because they often lack the financial and other resources needed to respond effectively.^12^ Unless carbon dioxide fertilization provides unexpected benefits, the overall effects of agricultural land conversion, climate change and reduced water availability could reduce China’s per-capita cereal production, compared with that recorded in 2000, by 18% by the 2040s.^24^ By 2030–2050, loss of cropland resulting from further urbanization and soil degradation could lead to a 13–18% decrease in China’s food production capacity compared with that recorded in 2005.^25^ The Asian Development Bank has predicted that as climate change proceeds, changes in precipitation and temperature will lead to droughts, severe storms and decreased agricultural productivity; subsidence will adversely affect freshwater fisheries, and increased temperatures will damage marine fisheries via ocean acidification and the loss of coral-reef nurseries.^26^

**Table 1:** Areas sown with major food crops and corresponding outputs and yields, China, 2013

Crop	2013 value**^a^**				Change compared with 2012 value^a^		
	Sown area, 1 000 hectares	Total output, 1 000 tonnes	Yield, kg/hectare		Sown area, 1 000 hectares	Output, 1 000 tonnes	Yield, kg/hectare
**All**	164 626.9	NA	NA		1 211.3	NA	NA
**Beans and peas**	9 223.6	1 595.3	1 730		−485.9	−135.3	−53
Soybean	6 790.5	1 195.1	1 760		−381.2	−109.9	−60
**Cereals**	93 768.6	55 269.2	5 894		1 156.2	1 334.5	71
Foxtail millet	715.7	174.6	2 440		−20.2	−5.0	NA
Maize	36 318.4	21 848.9	6 016		1 288.6	1 287.5	146
Rice	30 311.7	20 361.2	6 717		174.6	−62.4	−60
Early	5 804.4	3 413.5	5 881		39.5	84.4	106
Middle-season and one-crop late	18 186.3	13 297.6	7 312		167.6	−59.2	−101
Double-crop late	6 321.0	3 650.1	5 774		−32.4	−87.6	−108
Sorghum	582.3	289.2	4 965		−40.8	34.1	864
Wheat	24 117.3	12 192.6	5 056		−151.0	90.3	69
Other	1 723.2	402.7	2 337		−91.7	−9.6	65
**Tuber**	8 963.3	3 329.3	3 714		77.4	36.6	9
Potato	5 614.6	1 918.8	3 418		82.7	63.6	64

NA: not available.

^a^ All data from the *China agriculture yearbook 2014*.^20^

However, there are multiple uncertainties in the estimation of the effects of climate change on food supply. Any relationship between weather and crop yield is often crop- and area-specific and affected by differences in baseline climate, crop management, soil and the duration and timing of the crop’s exposure to various conditions. Rice yields have been found to be positively correlated with temperature in some areas of the world, but negatively correlated in others.^27^

The interaction between water resources and agriculture is likely to become increasingly important as climate changes. For example, crop productivity in China could be maintained or improved by irrigation – but only if the necessary water is available.^28^. Many of the 60 million people who live in the Mekong River basin are dependent in some way on the fisheries and aquaculture that are likely to be limited in the future – not only directly by climate change but also by changes in land use, flood mitigation, human population growth, increased off-take of water and overfishing.^29^^,^^30^

The impact of climate change is also likely to vary with the type of crop involved. For example, in the North China Plain, it has been projected that compared to the yields achieved in 1961–1990, maize yields will reduce by 9–10% during the 2020s, 16–19% during the 2050s and 25–26% in the 2080s.^31^ In contrast, it has been suggested that in the same area of China, yields of winter wheat may increase as a result of climate change.^32^

Continued or recurring food shortages pose a substantial threat to overall community health and well-being, social stability and human nutrition – especially the nutrition of children and other vulnerable groups. In China, much of the turmoil of past centuries and the destabilization of many great dynasties can be attributed to adverse climatic conditions that led first to food shortages and then to social disruption.^33^

Globally, about 2 billion people suffer from dietary iron and/or zinc deficiencies.^34^ Cereals and legumes that are so-called C_3_ plants – that is, plants that convert carbon dioxide and ribulose bisphosphate into 3-phosphoglycerate – provide these people’s main dietary source of iron and zinc. Such plants produce edible seeds with relatively low iron and zinc concentrations when grown, under field conditions, with the elevated atmospheric carbon dioxide concentrations that have been predicted for the middle of the 21st century.^35^ Elevations in the atmospheric concentration of carbon dioxide therefore threaten human nutrition.

Climate change poses a new challenge in the control of undernutrition in China – which is already a problem, especially in poor rural areas. Of the children younger than five years included in a national survey carried out in China in 2013, 8.1% were stunted, 2.4% were underweight and 1.9% were wasted.^36^

## Climate and water resources

Climate change is likely to have a major impact on global water resources by altering rainfall patterns and increasing the frequency of long and severe droughts in some areas, including China.^1^ Water tends to be abundant in the south of China but scarcer in the north. Many areas of the country lie in transition zones where water resources, and hence agricultural production, are already being reduced by changes in climate.^17^ Although in terms of the total volume of fresh water available in the country China is ranked sixth in the world, the amount of fresh water available per capita in China is only a quarter of the global mean value.^37^ The north of the country, which is similar in land area and population to the south, holds only 18% of the total fresh water despite having 65% of the total arable land. The Huanghe – or Yellow – and Yangtze Rivers are the two largest rivers in China. In the south, the Yangtze River has shown a small and statistically non-significant increase in annual run-off since 1960, driven by increasing precipitation. In the north, over the same period, the Huanghe River has shown persistent decline in run-off as the result of decreasing precipitation and heavy water use.^38^

As run-off is an important determinant of future crop yields, the Huanghe River’s declining run-off is already a cause for concern. It is difficult to estimate the effects of climate change on the future river flows in China for at least four reasons: (i) there are many uncertainties in regional climate projections and river management plans; (ii) glaciers will substantially influence water run-off in the future – but our capacity to predict the effects of melting glaciers is limited because only a few studies have addressed this issue; (iii) overextraction of water for irrigation, industrial and domestic usages is likely to increase as the human population grows and becomes increasingly wealthy; and (iv) no long-term standard socioeconomic development scenarios are available for China. If climate change impedes China’s agricultural production as projected,^39^ it will also undoubtedly threaten population health and sustainable development.

## Climate and health

China, with its fragile ecological systems, may be particularly vulnerable to the negative impacts of climate change. Climate change is likely to increase the frequency and intensity of weather events that are sufficiently extreme to have major impacts on China’s economy, environment and society.^22^ In 2001 a drought caused temporary shortages in drinking water for 33 million rural people and 22 million livestock and cost China an estimated  6.4 billion United States dollars (US$) in lost crop production.^40^ Similar events are known to have occurred in China’s history. Between 1585 and 1645, for example, China’s population may have declined by up to 40% due to a collapse of law and order and years of economic distress and warfare that were all underpinned by droughts and floods and associated outbreaks of famine and disease.^41^

The short-term, direct impact of climate change on health in China is likely to be due to increased air temperatures and air pollution, which have already substantially increased morbidity and mortality.^42^ Much of China, especially in the east, is affected by severe air pollution from fine particles that measure no more than 2.5 μm in aerodynamic diameter. A recent study projected future trends in the concentrations of such fine particulate matter and their short-term effect on mortality in eastern China.^43^ In this study, two distinct scenarios were modelled: a current legislation scenario, in which daily concentrations of fine particulate matter between 2005 and 2030 were limited by legislation in effect in 2005, and a maximum-reduction scenario that included the potential effects of the greatest reduction in concentration of fine particulate matter that was technically feasible in 2005. For each scenario, the likely changes in mortality attributed to the modelled air pollution were estimated using six population projections, two mortality rate projections, and the known relationships between mortality and air pollution. Under the current legislation scenario, the annual mean concentration of fine particulate matter was projected to decrease by 0.62 μg per m^3^ between 2005 and 2030 – a reduction that could avoid an estimated 124 000 deaths. The corresponding values recorded under the maximum-reduction scenario were a reduction of 20.41 μg per m^3^ and the prevention of at least 230 000 deaths.

China experienced extremely hot summers in 1988, 1990, 1994, 1998, 1999, 2002–2008 and 2013 and together, these resulted in thousands of excess deaths.^44^^,^^45^ Heatwaves can specifically increase morbidity and mortality among individuals with cerebrovascular, cardiovascular or respiratory system diseases. In China, 45% of all deaths are already attributed to cerebrovascular or cardiovascular diseases and the associated health-care costs and economic impacts of the losses in the labour force are estimated to total more than US$ 2500 million per year.^44^ In the absence of protective measures, this burden is likely to increase as mean air temperatures rise. In a study based on daily mortality and meteorological data collected in 66 Chinese communities between 2006 and 2011, 5% of the excess deaths were associated with heatwaves.^46^ In terms of the heatwave-associated mortality, certain groups, particularly individuals with cardiovascular, cerebrovascular or respiratory disease, the elderly and females, were found to be particularly vulnerable.

As well as full-blown heatwaves, climate change is also likely to increase the frequency of isolated hot days – further impairing health and productivity for millions of working people.^47^ Since high-temperature subsidies are allocated to Chinese employees for each day that they work in an extremely hot environment, hot days in China increase labour costs. The estimated mean annual cost of such subsidies between 1979 and 2005 was US$ 6.22 billion, about 0.2% of China’s gross domestic product.^47^ Assuming that the legislation on these subsidies remains unchanged throughout the 21st century, it has been estimated that as climate change proceeds, they will cost the country US$ 40.3 billion per year in the 2030s and US$ 161.1 billion per year by 2100.^47^

In the medium or long term, the largest effects of climate change on human health are likely to be indirect, for instance causing changes in the availability of food, shelter and water, decreased mental health and well-being and variations in the distribution and seasonality of infectious diseases.^48^ For example, climate change may have a major impact on malaria and other vector-borne diseases. When in a recent study the future distribution of malaria vectors and future changes in land use, urbanization and climate in China were modelled, the results indicated that by the 2030s, there would be a substantial increase in the size of the population exposed to the four dominant species of malaria vector in China.^49^

## Policy implications

This paper demonstrates that climate change may affect food, health systems and water in China in many ways. As globalization has linked China more closely with the rest of the world than ever before, attention should be paid to both the contribution of China to global warming and the effects of climate change on China. If China can rise up to tackle the challenges posed by climate change, its experience and success could provide useful guidance for other countries, particularly those in rapid transition. The world will encounter the consequences of climate change ever more clearly in the coming decades. It is encouraging to note that participants in the United Nations Framework Convention on Climate Change Conference that was held in Paris in late 2015 agreed to curb emissions of greenhouse gases and to try and keep global air temperatures no more than 2 °C above pre-industrial levels by the end of the 21st century. China has not only endorsed this agreement but has also issued its own so-called nationally determined contributions, which include several elements that if followed, should cause China’s carbon dioxide emissions to peak by 2030. These elements include an increase in the share of total primary renewable energy to 20% by 2030, a decrease in the carbon intensity of the gross domestic product to 60–65% of the 2005 level and an increase in China’s forest volume of 4.5 billion m^3^ – again relative to the 2005 value.^50^ If the proposed increase in forest stock can be achieved by sustainable reforestation that improves rural livelihoods and ecosystem services, then there may be co-benefits such as improvements in small-scale agricultural practices and local watershed management.

As no nation will be immune from the effects of a changing climate, concerted international commitment to reduce greenhouse gas emissions substantially at local, regional, country and global levels is still required.

## References

[1] Intergovernmental Panel on Climate Change. Climate change 2013: the physical science basis. Working Group I contribution to the Fifth Assessment Report of the Intergovernmental Panel on Climate Change. Cambridge: Cambridge University Press; 2013. Available from: http://www.climatechange2013.org/images/report/WG1AR5_ALL_FINAL.pdf [cited 2016 Apr 26].

[2] Intergovernmental Panel on Climate Change. Managing the risks of extreme events and disasters to advance climate change adaptation. Special report of the Intergovernmental Panel on Climate Change. Cambridge: Cambridge University Press; 2012. Available from: https://www.ipcc.ch/pdf/special-reports/srex/SREX_Full_Report.pdf [cited 2016 Apr 26].

[3] Tollefson J. China’s carbon emissions could peak sooner than forecast. Nature. 2016 3 24;531(7595):425–6. 10.1038/531425a 27008947

[4] The Lancet. Creating a healthier environment for the Chinese population. Lancet. 2016 3 26;387(10025):1252. 10.1016/S0140-6736(16)30063-0 27025418

[5] Bowles DC, Butler CD, Morisetti N. Climate change, conflict and health. J R Soc Med. 2015 10;108(10):390–5. 10.1177/0141076815603234 26432813 PMC4622275

[6] The state of food insecurity in the world 2001. Rome: Food and Agriculture Organization; 2002.

[7] Schmidhuber J, Tubiello FN. Global food security under climate change. Proc Natl Acad Sci USA. 2007 12 11;104(50):19703–8. 10.1073/pnas.0701976104 18077404 PMC2148361

[8] The state of food insecurity in the world 2015: strengthening the enabling environment for food security and nutrition. Rome: Food and Agriculture Organization; 2015.

[9] Butler CD. Revised hunger estimates accelerate apparent progress towards the MDG hunger target. Glob Food Secur. 2015;5:19–24. 10.1016/j.gfs.2015.04.002

[10] Butler CD. Famines, hunger, society and climate change. In: Butler CD, editor. Climate change and global health. Wallingford: CABI; 2014 pp. 124–34.

[11] Godfray HC, Pretty J, Thomas SM, Warham EJ, Beddington JR. Global food supply. Linking policy on climate and food. Science. 2011 2 25;331(6020):1013–4. 10.1126/science.1202899 21273449

[12] Knox J, Hess T, Daccache A, Wheeler T. Climate change impacts on crop productivity in Africa and South Asia. Environ Res Lett. 2012;7(3):034032 10.1088/1748-9326/7/3/034032

[13] Wheeler T, von Braun J. Climate change impacts on global food security. Science. 2013 8 2;341(6145):508–13. 10.1126/science.1239402 23908229

[14] Zhang Z, Duan Z, Chen Z, Xu P, Li G. Food security in China: the past, present and future. Plant Omics J. 2010;3:183–9.

[15] Tian H, Ren W, Tao B, Sun G, Chappelka A, Wang X, et al. Climate extremes and ozone pollution: a growing threat to China’s food security. Ecosyst Health Sustainability. 2016;2(1):e01203 10.1002/ehs2.1203

[16] Sun Y, Zhang X, Zwiers FW, Song L, Wan H, Hu T, et al. Rapid increase in the risk of extreme summer heat in eastern China. Nat Clim Chang. 2014;4(12):1082–5. 10.1038/nclimate2410

[17] Khan S, Hanjra M, Mu J. Water management and crop production for food security in China: a review. Agric Water Manage. 2009;96(3):349–60. 10.1016/j.agwat.2008.09.022

[18] Piao S, Ciais P, Huang Y, Shen Z, Peng S, Li J, et al. The impacts of climate change on water resources and agriculture in China. Nature. 2010 9 2;467(7311):43–51. 10.1038/nature09364 20811450

[19] Rulli MC, Saviori A, D’Odorico P. Global land and water grabbing. Proc Natl Acad Sci USA. 2013 1 15;110(3):892–7. 10.1073/pnas.1213163110 23284174 PMC3549107

[20] China agriculture yearbook 2014. Beijing: China Agriculture Press; 2015.

[21] Ye L, Tang H, Wu W, Yang P, Nelson G, Mason-D’Croz D, et al. Chinese food security and climate change: agriculture futures. Economics. 2014;8(2014-1):1–39. . 10.5018/economics-ejournal.ja.2014-1

[22] Intergovernmental Panel on Climate Change. Climate change 2014: impacts, adaptation, and vulnerability. Summary for policymakers. Working Group II contribution to the Fifth Assessment Report of the Intergovernmental Panel on Climate Change. Cambridge: Cambridge University Press; 2014. Available from: http://ipcc-wg2.gov/AR5/images/uploads/IPCC_WG2AR5_SPM_Approved.pdf [cited 2016 Apr 20].

[23] Huang J, Rozelle S. Agriculture, food security, and poverty in China. Past performance, future prospects, and implications for agricultural R&D policy. Washington: International Food Policy Research Institute; 2009. Available from: http://lib.icimod.org/record/14368/files/4068.PDF?version=1 [cited 2016 Jul 12].

[24] Xiong W, Conway D, Lin E, Xu Y, Ju H, Jiang J, et al. Future cereal production in China: modelling the interaction of climate change, water availability and socio-economic scenarios. Glob Environ Change. 2009;19(1):34–44. 10.1016/j.gloenvcha.2008.10.006

[25] Ye L, van Ranst E. Production scenarios and the effect of soil degradation on long-term food security in China. Glob Environ Change. 2009;19(4):464–81. 10.1016/j.gloenvcha.2009.06.002

[26] Addressing climate change risks, disasters, and adaptation in the people’s republic of China. Manila: Asian Development Bank; 2015.

[27] Zhang T, Zhu J, Wassmann R. Responses of rice yields to recent climate change in China: an empirical assessment based on long-term observations at different spatial scales (1981-2005). Agric Meteorol. 2010;150(7-8):1128–37. 10.1016/j.agrformet.2010.04.013

[28] Piao S, Ciais P, Huang Y, Shen Z, Peng S, Li J, et al. The impacts of climate change on water resources and agriculture in China. Nature. 2010 9 2;467(7311):43–51. 10.1038/nature09364 20811450

[29] Brander KM. Global fish production and climate change. Proc Natl Acad Sci USA. 2007 12 11;104(50):19709–14. 10.1073/pnas.0702059104 18077405 PMC2148362

[30] Ficke AD, Myrick CA, Hansen LJ. Potential impacts of global climate change on freshwater fisheries. Rev Fish Biol Fish. 2007;17(4):581–613. 10.1007/s11160-007-9059-5

[31] Tao F, Zhang Z, Liu J, Yokozawa M. Modelling the impacts of weather and climate variability on crop productivity over a large area: a new super- ensemble-based probabilistic projection. Agric Meteorol. 2009;149(8):1266–78. 10.1016/j.agrformet.2009.02.015

[32] Tao F, Zhang Z. Climate change, wheat productivity and water use in the North China Plain: a new super-ensemble-based probabilistic projection. Agric Meteorol. 2013;170(15):146–65. 10.1016/j.agrformet.2011.10.003

[33] McMichael AJ. Insights from past millennia into climatic impacts on human health and survival. Proc Natl Acad Sci USA. 2012 3 27;109(13):4730–7. 10.1073/pnas.1120177109 22315419 PMC3324023

[34] Tulchinsky TH. Micronutrient deficiency conditions: global health issues. Public Health Rev. 2010;32:243–55. 10.1186/s40985-017-0071-6 PMC5809998 29451564

[35] Myers SS, Zanobetti A, Kloog I, Huybers P, Leakey AD, Bloom AJ, et al. Increasing CO_2_ threatens human nutrition. Nature. 2014 6 5;510(7503):139–42. 10.1038/nature13179 24805231 PMC4810679

[36] Yu DM, Zhao LY, Yang ZY, Chang SY, Yu WT, Fang HY, et al. Comparison of undernutrition prevalence of children under 5 years in China between 2002 and 2013. Biomed Environ Sci. 2016 3;29(3):165–76. 27109127 10.3967/bes2016.021

[37] Cheng H, Hu Y, Zhao J. Meeting China’s water shortage crisis: current practices and challenges. Environ Sci Technol. 2009 1 15;43(2):240–4. 10.1021/es801934a 19238946

[38] Yang X, Xu J. Climate change and hydrological response: Recent studies on China’s river basins. In: Climate change and adaptation for water resources in Yellow River basin, China. Beijing: United Nations Educational, Scientific and Cultural Organization; 2010 pp. 24–37.

[39] Brown ME, Funk CC. Climate. Food security under climate change. Science. 2008 2 1;319(5863):580–1. 10.1126/science.1154102 18239116

[40] Shen C, Wang W, Hao Z, Gong W. Exceptional drought events over eastern China during the last five centuries. Clim Change. 2007;85(3-4):453–71. 10.1007/s10584-007-9283-y

[41] Wakeman FE Jr. The great enterprise: the Manchu reconstruction of imperial order in seventeenth century China. Volume 1 Berkeley: University of California Press; 1985.

[42] Kan H, Chen R, Tong S. Ambient air pollution, climate change, and population health in China. Environ Int. 2012 7;42:10–9. 10.1016/j.envint.2011.03.003 21440303

[43] Madaniyazi L, Nagashima T, Guo Y, Yu W, Tong S. Projecting fine particulate matter-related mortality in East China. Environ Sci Technol. 2015 9 15;49(18):11141–50. 10.1021/acs.est.5b01478 26226638

[44] Kurukulasuriya P, Zhao Y, Mao J, Guillemot J. Piloting climate change adaptation to protect human health in China. New York: Adaptation Learning Mechanism; 2016. Available from: http://www.adaptationlearning.net/project/piloting-climate-change-adaptation-protect-human-health-china-0 [cited 2016 May 3].

[45] Grieser J. Shanghai sets new all-time record (again) as heat wave bakes eastern China. The Washington Post. 2013 Aug 8. Available from: www.washingtonpost.com/news/capital-weather-gang/wp/2013/08/08/shanghai-sets-new-all-time-record-again-as-record-heat-bakes-eastern-china/ [cited 2016 May 3].

[46] Ma W, Zeng W, Zhou M, Wang L, Rutherford S, Lin H, et al. The short-term effect of heat waves on mortality and its modifiers in China: an analysis from 66 communities. Environ Int. 2015 2;75:103–9. 10.1016/j.envint.2014.11.004 25461419

[47] Zhao Y, Sultan B, Vautard R, Braconnot P, Wang HJ, Ducharne A. Potential escalation of heat-related working costs with climate and socioeconomic changes in China. Proc Natl Acad Sci USA. 2016 4 26;113(17):4640–5. 10.1073/pnas.1521828113 27044089 PMC4855586

[48] Watts N, Adger WN, Agnolucci P, Blackstock J, Byass P, Cai W, et al. Health and climate change: policy responses to protect public health. Lancet. 2015 11 7;386(10006):1861–914. 10.1016/S0140-6736(15)60854-6 26111439

[49] Ren Z, Wang D, Ma A, Hwang J, Bennett A, Sturrock HJ, et al. Predicting malaria vector distribution under climate change scenarios in China: challenges for malaria elimination. Sci Rep. 2016 2 12;6:20604. 10.1038/srep20604 26868185 PMC4751525

[50] Zheng T, Zhu J, Wang S, Fang J. When will China achieve its carbon emission peak? Natl Sci Rev. 2016;3(1):8–12. 10.1093/nsr/nwv079

